# Lateral diffusion of single polymer molecules at interfaces between water and oil†

**DOI:** 10.1039/d0ra02630a

**Published:** 2020-04-27

**Authors:** Zhuo Li, Jingfa Yang, Javoris V. Hollingsworth, Jiang Zhao

**Affiliations:** Beijing National Laboratory for Molecular Science, Institute of Chemistry, Chinese Academy of Sciences Beijing 100190 China yangjf@iccas.ac.cn jzhao@iccas.ac.cn; University of Chinese Academy of Sciences Beijing 100049 China; Department of Chemistry and Biochemistry, University of St. Thomas Houston Texas 77006 USA

## Abstract

Lateral diffusion of polymer molecules at the interfaces between immiscible oil and water is investigated at the single molecular level. The interfaces between water and alkanes are chosen as the model systems and polyethylene oxide (PEO) is the model polymer. Fluorescence correlation spectroscopy is used to measure the interfacial diffusion of fluorescence-labeled PEO with its molecular weight ranging over more than an order of magnitude. It is discovered that the interfacial diffusion coefficient scales with the molecular weight by the exponent of −0.5. Detailed analysis shows that the PEO chain takes an ideal two-dimensional random coil conformation at these fluidic interfaces and the bigger contribution from water's hydrodynamic friction is discovered.

## Abstract 2

A new scaling law is discovered for polymer lateral diffusion at water/oil interfaces.

## Graphical abstract

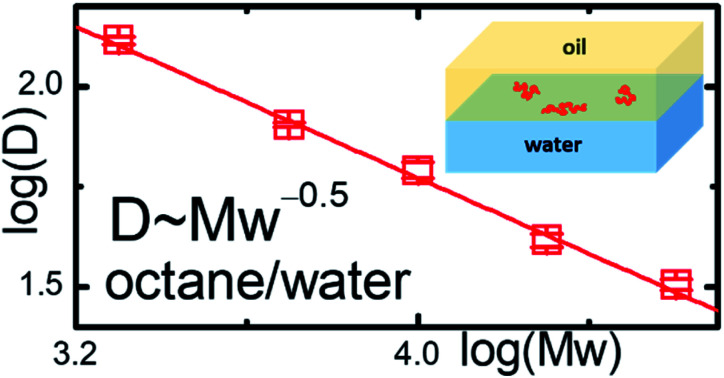

## Introduction

It is commonly observed in nature that aqueous solutions meet hydrophobic substances and many processes such as biochemical reactions occur at such interfaces.^1^ The interfaces formed between water and oil widely exist in our daily life and play crucial roles in many fields, such as environmental science and engineering,^2^ petrochemical industries,^3^ food industries,^4^ biological systems^5,6^ and many other areas and fields.^7,8^ Understanding the structure and dynamics of oil–water interfaces is important not only for scientific but also for practical reasons – for example, it can help to develop more efficient medicines due to the crucial role of such fluidic interfaces at which medicine molecules interact with biomacromolecules.^9^

Dynamics of macromolecules at the interfaces determines the reaction kinetics *via* the mass transportation process and in general, the interfacial dynamics exhibits remarkable differences compared with bulk solutions or melts. The differences related to such two-dimensional or semi-two-dimensional processes are believed to depend on a number of factors, such as chain conformation, interfacial viscosity, surface heterogeneity, interfacial interactions, *etc.* Pioneering studies have been conducted on the interfacial diffusion of macromolecules along fluidic membrane and solid–liquid interfaces,^10–19^ in which the interfacial diffusivity exhibits scaling laws with the molecular weight. For DNA molecules diffusing on supported fluidic lipid membrane, Rouse dynamics was discovered with the diffusion coefficient having an inverse linear scale law.^10,11^ For synthetic polymers on liquid–solid interfaces, reptation dynamics having a nonlinear scaling law was discovered,^12,13^ and also depending on the chemical and topological nature of the solid surface, the Rouse dynamics can be recovered.^14^ Compared with the liquid–solid interfaces, the lateral diffusion of polymer molecules along liquid–liquid interfaces has been less investigated.^15,16^ The diffusion at liquid–liquid interfaces should differ largely to the solid–liquid interface because of the thermal-activation from both liquid phases, making the hydrodynamics an important role. Due to the different strength of interaction between the chain molecules with the liquids, different contributions of hydrodynamics from the two liquids can be different and have a big effect to the interfacial dynamics. All these issues have not been well-investigated although they are certainly interesting and important topics, making it significant to investigate lateral diffusion at the liquid–liquid interface.

In the current study, the lateral diffusion of polymer molecules at the water–oil interface is investigated at single molecular level using fluorescence correlation spectroscopy (FCS). The water–alkane interface is chosen as the model system, because both water and alkane with low carbon number are ideal liquids and no dynamical heterogeneity is introduced, making their interface a perfect system to investigate. Fluorescence-labeled linear polyethylene oxide (PEO) is chosen as the model molecule, which behaves as an amphiphilic molecule diffusing at the interface. The high sensitivity and spatial resolution of FCS enable precise measurements of interfacial diffusion rate of individual fluorescence-labelled polymer.^12–15,20–25^ A new scaling law of interfacial diffusion rate with the molecular weight is discovered, providing important evidence for the understanding of the interfacial molecular structure and diffusion mechanism of the polymer.

## Results and discussions

**Fig. 1:**
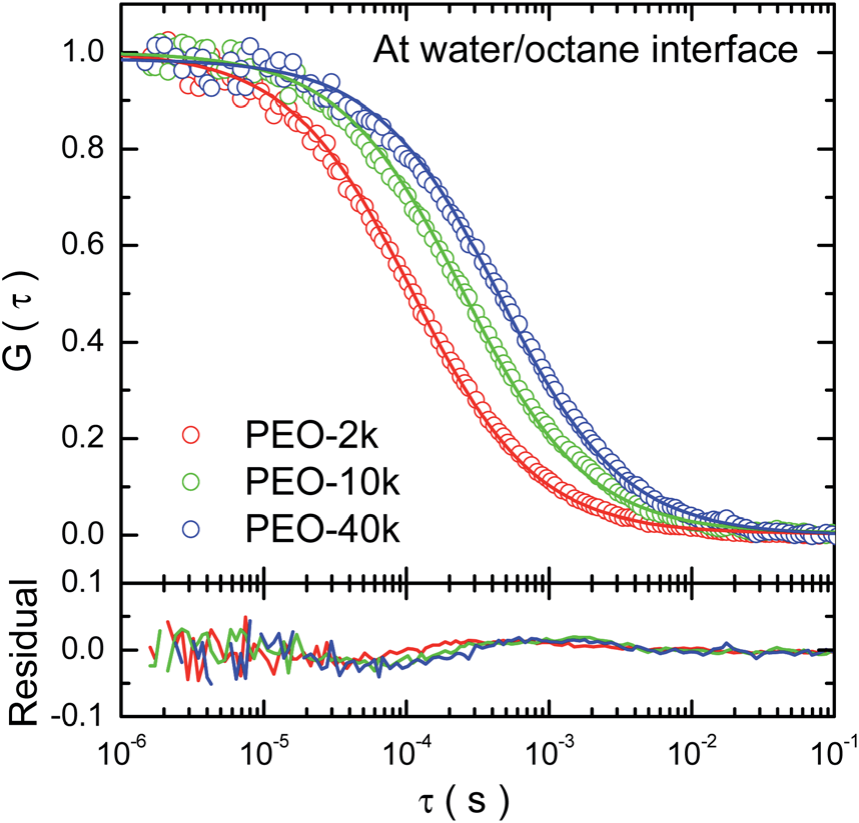
Typical normalized autocorrelation functions of fluorescence-labeled PEO molecules diffusing at the octane–water interface. The solid curves denote numerical fitting using two-dimensional Brownian motion model. The residuals showing the difference between the data and fitting by two-dimensional Brownian motion are provided.

Typical auto-correlation function data of fluorescence-labeled PEO molecules diffusing at octane–water interfaces are displayed in Fig. 1. The data are fitted by the two-dimensional Brownian motion model expressed as *G*(*τ*) = (π*w*_0_^2^〈*ρ*〉)^−1^(1 + 4*D*_s_*τ*/*w*_0_^2^)^−1^, where *D*_s_ is the diffusion coefficient, *w*_0_ the lateral radius of the confocal volume and 〈*ρ*〉 the average number of fluorescent molecules inside the confocal volume, *i.e.* the projected area of this volume on the interfacial plane.^20,21^ The residuals of the fitting are plotted in the sample figure. The mean square displacement (MSD) data are calculated from the auto-correlation functions^26,27^ and the numerical fittings using normal diffusion model agree with the data (detailed in ESI†). All of these facts indicate that the lateral diffusion of PEO at the alkane/water interfaces is proved to be Brownian.

**Fig. 2:**
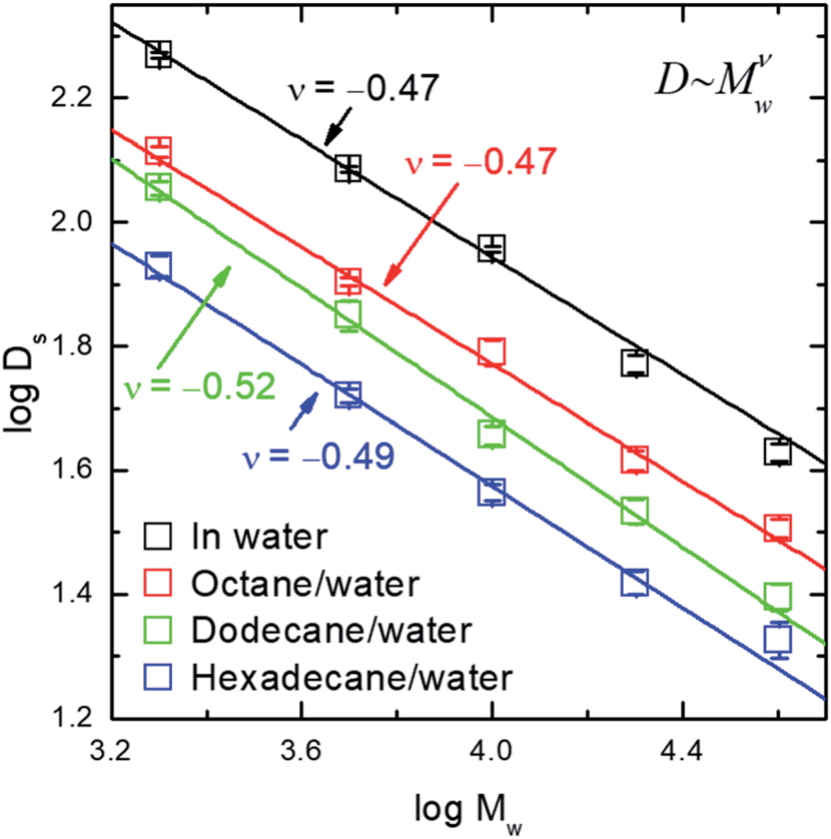
The logarithm of interfacial diffusion coefficient, *D*_s_, of PEO (in unit of μm^2^ s^−1^) as a function of the logarithm of molecular weight (*M*_w_) in the unit of g mol^−1^.

The interfacial diffusion coefficient of PEO at different alkane/water interfaces as a function of the molecular weight (*M*_w_) is displayed in Fig. 2, with the data of diffusion coefficient of PEO in water solution displayed for comparison. Other auto-correlation function data are shown in ESI (Fig. S3†).

Three features are noticed. (1) The values of interfacial diffusion coefficient (*D*_s_) of PEO at all alkane/water interfaces are lower than those in the bulk water solution. This is a clear indication of the interfacial diffusion as one degree of freedom of the molecule is restricted and the thermo-activation in that dimension is missing, resulting in a slower diffusion rate. One subtle point is whether the adsorption is because of the fluorescent molecules at the chain end and not because of PEO chain itself. Control experiments measuring fluorescence intensity at the interface show that the fluorescence intensity using labelled PEO is 60 times of that using free labels alone, although the latter has a higher concentration in solution. The details are shown in ESI (Fig. S4†). This has excluded the possibility of adsorption brought by fluorescence-labeling. (2) All *D*_s_ values of PEO decrease with the increase of the molecular weight. The data demonstrate a scaling law of *D*_s_ ∼ *M*_w_^−0.5^ at all three interfaces. (3) For PEO of identical molecular weight, the *D*_s_ values are lower at the interface between water and alkane with higher carbon number, as a result of less activation from the more viscous liquid.

The scaling exponent index of −0.5 reveals a new scaling law of interfacial diffusion of polymer, besides the two important cases ever reported, *i.e.* the scaling index of −1 for Rouse dynamics^10,11,17^ and scaling index of −1.5 for reptation dynamics.^12–14^ This indicates a different mechanism of diffusive motion at the interface between two immiscible liquids. For the DNA molecule residing on the top of lipid membrane, it experiences the friction exerted on each of its repeating unit and therefore its diffusion rate has an inverse-linear scaling law with molecular weight.^10^ For polymer chain adsorbed on solid–liquid interfaces, the polymer chain moves along its backbone and experiences the friction nonlinearly dependent on the degree of polymerization.^12^ In the current case, the PEO chain is sandwiched between two fluidic phases, where the hydrodynamics should have a strong effect. In this case, the diffusion mechanism can be understood in a way similar to the Rouse–Zimm model – the chain is regarded as an object with solvent trapped inside its coil.^28,29^ The current result is different to a previous report of polymer diffusion at an interface between water and a polymeric liquid, in which a scaling index of −0.6 was found and the mechanism of desorption-mediated diffusion was proposed.^16^

The PEO molecule adsorbed at water/alkane interface is considered to have its segments distributed in the two-dimensional space and therefore to be treated as a disk-like or pancake-like object. Theoretically, the lateral diffusion coefficient of a circular disk-like object can be expressed by *D*_s_ = 3*k*_B_*T*/16*R*(*η*_1_ + *η*_2_), where *R* is the radius of the disk, *η*_1_ and *η*_2_ are the viscosity of the two liquids, respectively.^30,31^ The *D*_s_ value is inversely proportional to the disk's radius. More specifically, it has been developed theoretically that the lateral interfacial diffusion coefficient of a chain molecule residing in a two-dimensional fluid is inversely proportional to its two-dimensional radius of gyration (*R*_g_), by the expression of 
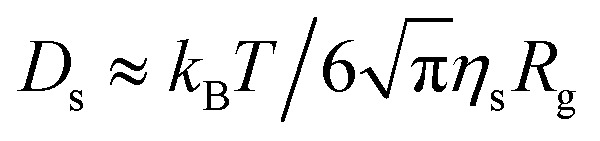
, where *η*_s_ denotes the interfacial viscosity defined to have the average value of the viscosity of two immiscible liquid phases, *i.e. η*_s_ = (*η*_1_ + *η*_2_)/2.^5,32,33^ By comparing this theoretical result and the current experimental data, it is shown that at the current interfaces formed between the alkanes and water, the two-dimensional radius of gyration of PEO and its degree of polymerization (*N*) has a relation of *R*_g_ ∼ *N*^0.5^. This result demonstrates that the PEO molecule takes an ideal two-dimensional random coil conformation at the interface between water and alkanes (*n*-octane, *n*-dodecane and *n*-hexadecane), in contrast to 0.75 scaling law of the swelled coil in two-dimension.^28,29^

The reason why PEO molecule adopts a two-dimensional random coil conformation at the alkane/water interface is attributed to the negative contribution to exclusive volume by alkanes. Water serves as the good solvent of PEO while alkanes are non-solvent. Research has shown the interaction between water molecule and the oxygen atom in the ether group dominates the solubility of PEO in water.^34^ At the water/alkane interface, the PEO molecule are brought close to alkane molecules, which can weaken the water–oxygen attraction and put a negative contribution to the exclusive volume. This can result in a zero (or near zero) exclusive volume, leading to a two-dimensional random coil.

The comparison between the calculated values of interfacial diffusion coefficient according to the two-dimensional random coil model with the experimentally measured values show the relative weighing of the contributions from the two fluid phase. Taking the published data of PEO,^29^ the values of *R*_g_ and therefore the interfacial diffusion coefficient are calculated, as displayed in Table 1. The calculated values of interfacial diffusion coefficient (*D*_cal-1_) are close to the measured ones (*D*_exp_) of dodecane/water interface only while the *D*_cal-1_ values are considerably higher than *D*_exp_ for octane/water and considerably lower for hexadecane/water case. Considering the fact that the difference in viscosity between three alkanes and water – the viscosity of dodecane (1.480 mPa s) is close to water (1.005 mPa s) while that of octane (0.543 mPa s) is lower and that of hexadecane (3.547 mPa s) is higher than water, it is reasoned that the interfacial viscosity experienced by the PEO molecule differs from the mere average value of the viscosity of the water and alkane, *i.e.* the water phase weighs more than the alkanes. Therefore, a weighing factor is introduced into the estimation as *η*_s_ = *xη*_1_ + (1 − *x*)*η*_2_, where *χ* is the weighing factor of the viscosity of liquid 1, set to be water here. By taking *χ* as 0.8, the calculated values of interfacial diffusion coefficients (*D*_cal-2_) agree with the measured values (Table 1). These results indicate a bigger contribution to the interfacial hydrodynamic friction from the water than from alkanes, attributed to the stronger attraction between the PEO chains and water. This is further supported by the weak dependence of the *D*_s_ values on the viscosity of the alkane phase, as detailed in ESI.†

**Table 1:** The comparison between the calculated and measured values of interfacial diffusion coefficient^a^

*M*_w_ (×10^3^ g mol^−1^)	Octane/water			Dodecane/water			Hexadecane/water		
	*D*_exp_	*D*_cal-1_	*D*_cal-2_	*D*_exp_	*D*_cal-1_	*D*_cal-2_	*D*_exp_	*D*_cal-1_	*D*_cal-2_
2.0	130.1	164.5	139.5	113.6	102.5	115.8	85.0	57.2	86.0
5.0	80.1	104.1	88.3	70.7	64.8	73.2	52.4	36.2	54.4
10.0	61.6	73.6	62.4	45.3	45.8	51.8	36.7	25.6	38.5
20.0	41.3	52.0	44.1	34.2	32.4	36.6	26.2	18.1	27.2
40.0	32.0	36.8	31.2	24.8	22.9	25.9	21.1	12.8	19.2

a *D*_exp_ is the value of interfacial diffusion coefficient measured by FCS. The diffusion coefficient is unit of μm^2^ s^−1^. *D*_cal-1_ and *D*_cal-2_ are the calculated values using the weighing factor for water contribution of 0.5 and 0.8, respectively.

## Experimental

### Construction of alkane–water interfaces

All *n*-alkanes (*n*-octane, *n*-dodecane, and *n*-hexadecane) with purity of ≥99% were purchased from Sigma-Aldrich. Prior to use, alkanes were purified several times by column chromatography using a basic alumina stationary phase.^35,36^ Control experiments measuring interfacial tension demonstrate that purification of alkanes is an essential step to keep the proper interfacial properties, whose interfacial tension can be maintained constant for long enough time, as detailed in ESI (Fig. S1†).

### Fluorescence-labelled PEO

Amino-terminated PEO (PEO-NH_2_) with weight-average molecular weights (*M*_w_) of 2.0, 5.0, 10.0, 20.0 and 40.0 kg mol^−1^ were purchased from Nanocs (USA). Polydispersity indexes (*M*_w_/*M*_n_) of these PEO samples are in the range of 1.03–1.07 (data from the provider). A fluorescent molecule, Rhodamine 6G (Rh6G), was chemically connected to the PEO chain end by carboxylation *via* condensation reaction between the amine group of PEO and carboxyl group of Rh6G. The sample was further purified by size exclusion chromatography for multiple times to remove free Rh6G molecules, using swollen P-6 Media (Bio-Gel, USA) as a stationary phase and water as a mobile phase. After purification, the labelled PEO was collected, freeze-dried and stored at −20 °C for future use.

### Fluorescence correlation spectroscopy measurements

FCS measurements were conducted using a commercial system (LSM780, Carl Zeiss, Germany) with an inverted microscope. A water-immersion objective lens (C-Apochromat, 40×, numerical aperture = 1.2) was used and the 514 nm output of an Argon ion laser was chosen as the excitation light. The principle of FCS has been described multiple times previously and are not detailed here.^35^ The schematic diagram of FCS measurements in this study is shown in ESI (Fig. S2†). In the current study, the excitation-detection volume was calibrated using free Rh6G in aqueous solution at a concentration of 5 × 10^−9^ M, taking its diffusion coefficient of 360.5 μm^2^ s^−1^ at 20 °C.^37^ It was determined that lateral radius of the confocal volume is 0.24 μm and the vertical half-length is 1.20 μm.

Water–alkane interfacial systems were prepared in a sample cell constructed using a cylinder-quartz cuvette with a 0.17 mm-thick microscope coverslip glued at the bottom. All sample cells were thoroughly cleaned with a significant amount of deionized water and the treatment by oxygen plasma, respectively. The preparation of the liquid–liquid interface was done by adding deionized water (300–400 μL) and purified alkane (800–1000 μL) into the sample cell, successively. Afterwards, approximately 1–5 μL aqueous solution of fluorescence-labelled PEO (∼1 × 10^−9^ M) was injected into the water phase inside the sample cell, which was later sealed at the top. The sample was incubated for more than 8 hours before FCS measurements. Later measurements have shown that the final surface concentration of PEO is 2.1–17.8 molecule per μm^2^, corresponding to the average inter-molecular distance of 140–390 nm.

To conduct FCS measurements at the interface, the objective lens was adjusted so that the focal point is at the water–alkane interface when maximum photon counts were recorded – the contrast was very sharp as the photon counts dropped to background noise level if the focal point was moved away from the interfacial region, *i.e.* into the bulk water or bulk alkane phase. For each sample, 10–20 independent measurements were performed at different locations at the interface. For each experimental condition, the experiments were repeated more than three times on different days using fresh samples.

## Conclusions

At the interfaces between two immiscible fluids – water and alkane of small carbon number, the amphiphilic PEO chain diffuses laterally, experiencing hydrodynamic drags from both phases. The absolute values of interfacial diffusion coefficients demonstrate a bigger contribution from the hydrodynamics from the water phase, attributed to a stronger attraction between water and PEO molecules. The PEO chain takes a two-dimensional random coil conformation, as demonstrated by the scaling law of its interfacial diffusion coefficient with the degree of polymerization, *D*_s_ ∼ *N*^−0.5^.

## Conflicts of interest

There are no conflicts to declare.

## Supplementary Material

RA-010-D0RA02630A-s001
