# Supplementary material for: Lateral diffusion of single polymer molecules at interfaces between water and oil
Source: RSC Adv. 2020 Apr 27;10(28):16565–9. doi: 10.1039/d0ra02630a (PMC9052869; doi:10.1039/d0ra02630a)
Supplement: RA-010-D0RA02630A-s001 [file RA-010-D0RA02630A-s001.pdf]

Electronic Supplementary Material (ESI) for RSC Advances.
This journal is © The Royal Society of Chemistry 2020

Supporting Information of "Lateral Diffusion of Single Polymer Molecules at Interfaces between Water and Oil"

Zhuo Li,[a,b] Jingfa Yang,[*,a,b] Javoris V. Hollingsworth,[c] Jiang Zhao[*,a,b]

[a] Beijing National Laboratory for Molecular Science, Institute of Chemistry, Chinese Academy of Sciences, Beijing 100190, China

[b] University of Chinese Academy of Sciences, Beijing 100049, China

[c] Department of Chemistry and Biochemistry, University of St. Thomas, Houston, Texas 77006, USA

1. *The effect of purification of the alkanes to the stability of the interfaces*

All n-alkane samples used in the experiments (n-octane, n-dodecane, n-hexadecane) with purity of ≥99% were purchased from Sigma-Aldrich. De-ionized water with a resistivity of 18.2 MΩ·cm was obtained from a Milli-Q water purification system. Prior to use, alkanes were purified by column chromatography using a basic alumina stationary phase for several times to remove amphiphilic impurities. Purification of alkanes has been shown essential for the construction of the stable interface designed, as tracer amount of impurities can adsorb to the interface and consequently affects interfacial properties.

The interfacial tension of the two phases was measured using Wihelmy plate method (Tensiometer K100, Kruss, Germany). The platinum plate was cleaned with a flame before every measurements. The measurements of the interfacial tension for each alkane-water system was performed for more than 20 minutes. Figure S1 shows the value of interfacial tension of three alkane/water system as

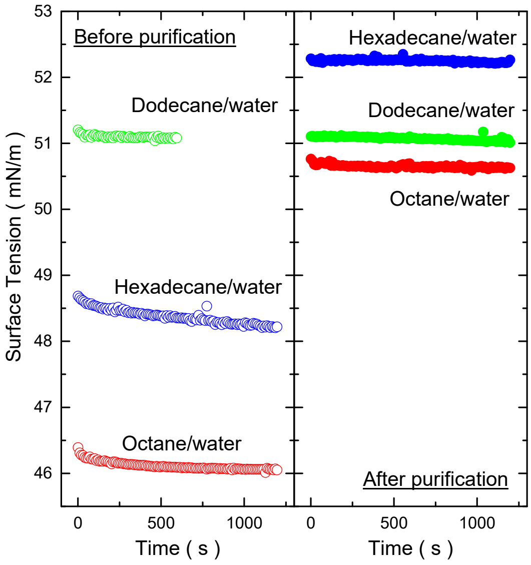

Figure S1 The values of interfacial tension as a function of time for three alkane/water systems, before and after purification.

* Corresponding author: yangjf@iccas.ac.cn and jzhao@iccas.ac.cn.

a function of time. It is immediately noticed that after purification, the values of interfacial tension of n-octane and n-hexadecane become considerably higher and the values of three alkanes exhibit a right order. Also, the noticeable decay of interfacial tension with time before purification is largely suppressed by purification.

2. *The experimental setup of the fluorescence correlation spectroscopy for liquid-liquid interfaces*

   Figure S2 shows the schematic of the FCS setup together with the sample cell for the measurements of interfacial diffusion at liquid-liquid interface.

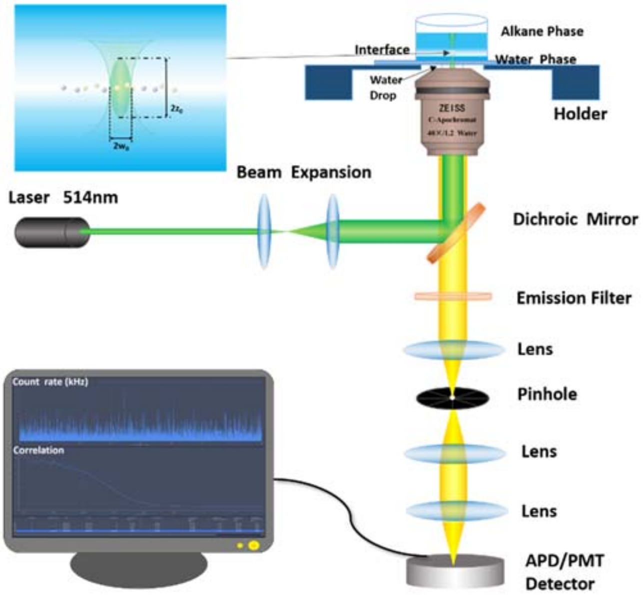

Figure S2 The schematic diagram of fluorescence correlation spectroscopy setup for the measurements of lateral diffusion of PEO molecules at the alkane-water interfaces.

3. *The auto-correlation function of PEO diffusion at alkane/water interfaces*

   The typical normalized auto-correlation functions of diffusion of fluorescence-labeled PEO at alkane/water interfaces are provided in Figure S3, in which it is observed that the diffusion of PEO with larger molecular weight diffuses slower than the sample of smaller molecular weight. As an example, the PEO sample with the molecular weight of 5.0 kg mol^{-1} at interfaces between water and three alkanes are shown, telling that the interfacial diffusion is slower at interfaces formed between water and alkane with bigger carbon number.

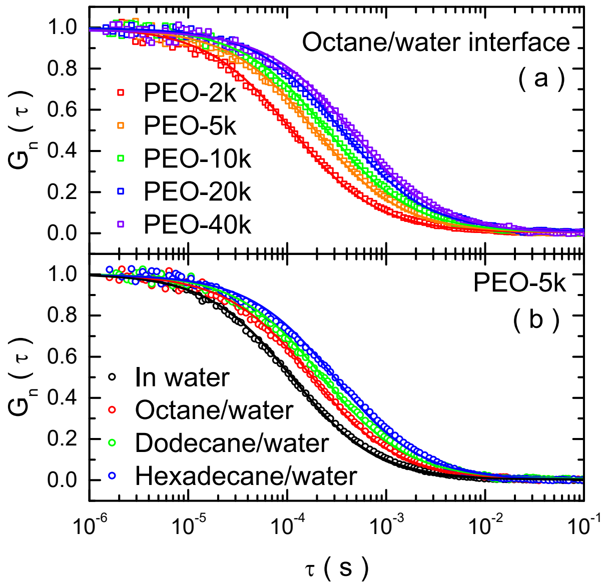

Figure S3 (a) Normalized autocorrelation functions of diffusion of PEO with different molecular weight at the octane/water interface. (b) The data of PEO (M_w = 5.0 kg mol^{-1}) diffusing at different alkane-water interfaces. The data of PEO's diffusion in water solution is also shown as a comparison. The solid lines denote the results of numerical fitting using Brownian motion model.

4. *Evidence to prove that the adsorption at the interface alkane/water interface is by PEO molecules*

Control experiments were conducted to check that the adsorption of PEO chain at alkane/water interface is by PEO molecules to exclude the possibility of adsorption induced by the fluorescent molecule attached to the chain end. The fluorescence intensities at the dodecane/water interface was measured by using

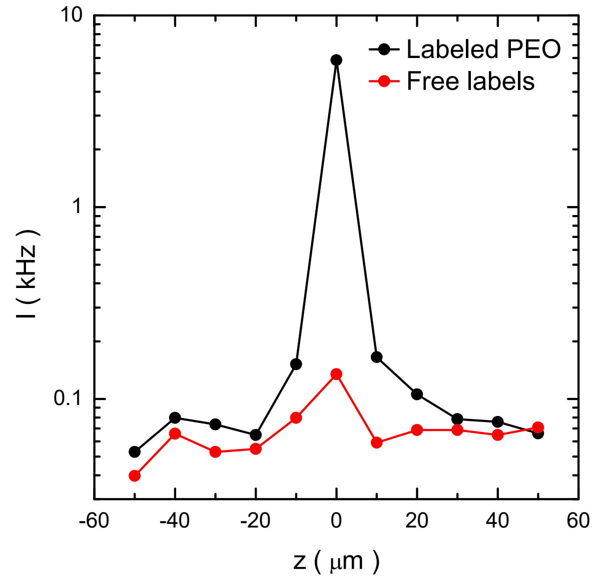

Figure S4 Fluorescence intensity (i as the photon counts) of labeled PEO (black) and free labels (red) at octane/water interface (at the position of z = 0 μm).

two samples separately: PEO labeled with Alexa 488 fluorophore and the free Alexa 488 molecules along. Alexa-488 was chosen because of its hydrophilicity, which prevent its adsorption to the alkane/water interface. In these experiments, the concentration of free Alexa 488 was twice of that of labeled PEO. The data of the fluorescence intensities at the interface are shown in Figure S4. The data show that the intensity from the labeled PEO sample is ~50 times higher than that of free fluorophores, indicating that the adsorption is by PEO molecule itself.

5. *The dependence of interfacial diffusion coefficient on interfacial viscosity*

Figure S5a shows the diffusion coefficient (D_s) of PEO at the interface between water and alkanes plotted as a function of the viscosity of the alkane phase (η_{oil}).

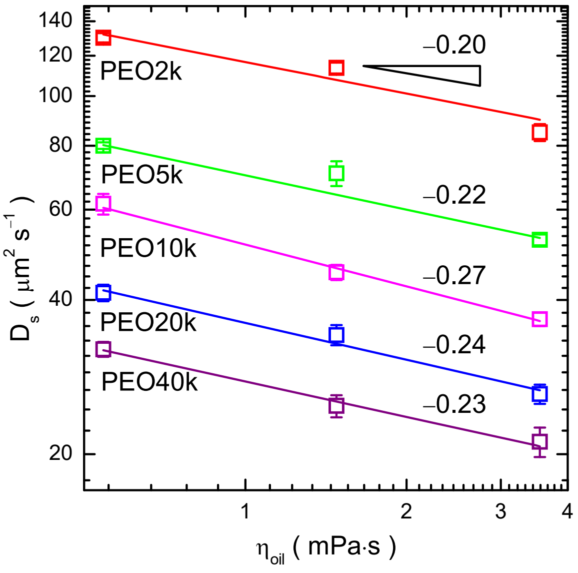

Figure S5 Lateral diffusion coefficient (D_s) of PEO at the interfaces between water and octane, dodecane and hexadecane as a function of the viscosity of the alkane phase (η_{oil}).

6. *Proof of Brownian motion of the PEO at water/alkane interfaces*

The data of mean square displacement, $\left\langle r(\tau)^2 \right\rangle$, of PEO molecule at alkane/water interfaces prove the lateral diffusion is Brownian motion. Figure S6 shows MSD calculated from auto-correlation functions of PEO molecules diffusing on octane/water interface.[1,2] The solid lines show the results of fitting by the expression of $\left\langle r(\tau)^2 \right\rangle = 4D\tau^{\alpha}$, in which D and τ are diffusion coefficient and time lag, respectively, and α is the anomalous index. The fitting results show that the α value is close to 1.0, indicating the absence of anomalous diffusion and the motion of PEO molecule at the interfaces is Brownian.

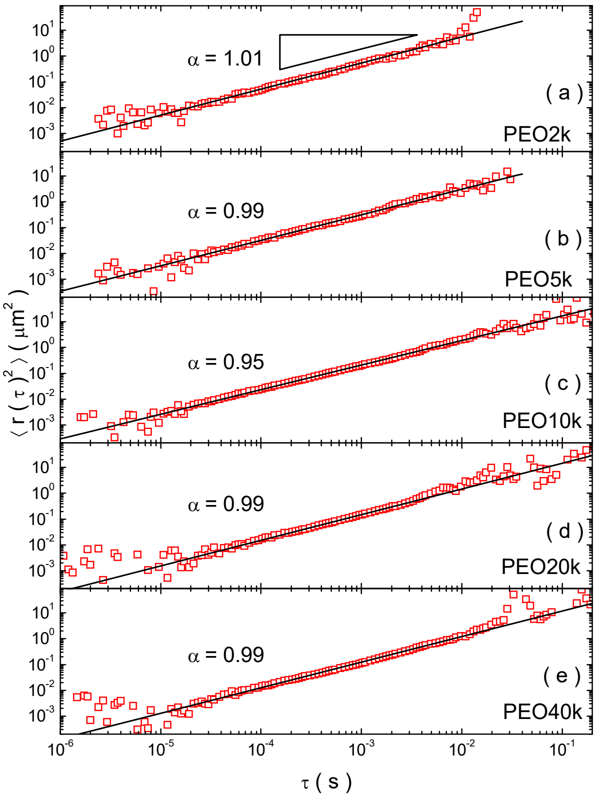

Figure S6 Data of mean square displacement of PEO molecules diffusing at the interface between octane and water, calculated from the auto-correlation function. The solid lines denote the fitting results by $\left\langle r(\tau)^2 \right\rangle = 4D\tau^{\alpha}$. The anomalous index ($\alpha$) of each data set is displayed, showing the absence of anomalous diffusion.

References

1. Feng, L. G.; Yang, J. F.; Zhao, J.; Wang, D. P.; Koynov, K.; Butt, H. J., Fluorescence correlation spectroscopy of repulsive systems: Theory, simulation, and experiment. *J. Chem. Phys.* **2013**, *138*, 214902.

2. Stolle, M. D.; Fradin, C, Anomalous Diffusion in Inverted Variable-Lengthscale Fluorescence Correlation Spectroscopy. *Biophys. J.* **2019**, *116*, 791-806.
